# A Rare Case of Shone Complex With a Persistent Left Superior Vena Cava and Absent Right Superior Vena Cava

**DOI:** 10.7759/cureus.85658

**Published:** 2025-06-09

**Authors:** Balaji Aironi, Gaurish Sawant, Palakkumar P Shah, Hitesh Makhija, Rajat Lohiya

**Affiliations:** 1 Cardiovascular and Thoracic Surgery, King Edward Memorial Hospital and Seth Gordhandas Sunderdas Medical College, Mumbai, IND; 2 Surgery, Maulana Azad Medical College, New Delhi, IND

**Keywords:** absent right svc, congenital heart disease, persistent left svc, shone complex, supravalvular mitral ring

## Abstract

Shone complex is a rare and underrecognized congenital heart disease involving four obstructive lesions of the left heart. This report presents a rare case of incomplete Shone complex, along with other associated anomalies, requires an extensive clinical workup for accurate diagnosis and appropriate surgical planning.

## Introduction

Shone complex is a rare congenital heart condition characterized by multiple obstructive lesions affecting the structures on the left side of the heart [1]. However, incomplete forms are more commonly encountered, particularly among older individuals. In Central India, the prevalence of Shone complex in the general pediatric population is only 0.9% [2]. A persistent left superior vena cava (PLSVC) accompanied by an absent right superior vena cava (SVC) is an even rarer congenital anomaly. The condition exhibits considerable anatomical and physiological variation, ranging from mild cases that require no intervention to severe cases necessitating surgical correction. Long-term prognosis in severe cases is generally poor, with reported mortality rates of 24-27% [3,4]. This report presents a case of a 13-year-old boy diagnosed with Shone complex, PLSVC, and an absent right SVC, who underwent successful surgical treatment.

## Case presentation

A 13-year-old boy presented with dyspnea on exertion, accompanied by sweating and giddiness, persisting for two weeks. Although he had experienced symptoms for the past five years, he sought medical attention only after a noticeable progression in severity. On examination, his pulse rate was 88 beats per minute, and his blood pressure was 100/60 mmHg. A heaving apical impulse was palpable in the fifth intercostal space, 2 cm lateral to the midclavicular line. A grade 3/6 ejection systolic murmur with a crescendo-decrescendo character was audible in the aortic area and radiated to both carotid arteries. Additionally, a grade 2/6 pansystolic murmur was heard in the mitral area.

Electrocardiography revealed a normal sinus rate and rhythm, with evidence of left ventricular hypertrophy accompanied by a strain pattern. Chest radiography demonstrated a cardiothoracic ratio of 0.6, straightening of the left heart border, and signs suggestive of pulmonary hypertension.

Echocardiography showed a left atrial dimension of 52 mm (Z score=4.16) × 66 mm ( Z score=5.33) and an aortic annulus measuring 17 mm (Z score = -0.93). The left ventricular internal dimensions were 42 mm (Z score=5.21) in systole and 50 mm (Z score=2.26) in diastole. The thickness of the left ventricular posterior wall and interventricular septum measured 14 mm (Z score=5.46) and 15 mm (Z score=3.38), respectively, indicating concentric hypertrophy. The ejection fraction was 25%, with global left ventricular hypokinesia and severe LV dysfunction. A subaortic membrane was noted, attached to the right coronary cusp, resulting in severe aortic stenosis (peak velocity=5.32 m/s; mean gradient=112/70 mmHg) and moderate aortic regurgitation (pressure half-time=252 ms). A supramitral membrane with an area of 1.2 cm² and a transmitral gradient of 17/9 mmHg was also present, along with mild mitral regurgitation. Severe tricuspid regurgitation was observed, with a tricuspid annulus measuring 38 mm and severe pulmonary hypertension (pulmonary artery systolic pressure=60 mmHg, estimated by tricuspid regurgitation jet). No evidence of coarctation of the aorta was found.

CT aortography revealed mild dilation of the ascending aorta extending to the proximal arch (26 mm), with a normal appearance of the remaining arch and descending aorta. A membrane measuring 1.2 mm in thickness and approximately 4 mm in width was identified at the level of the aortic annulus, along with leaflet thickening and severe aortic stenosis. After completion of the preoperative workup and confirmation of anesthetic fitness, the patient was scheduled for excision of the aortic and mitral membranes, along with intraoperative assessment of the aortic, mitral, and tricuspid valves.

**Figure 1:**
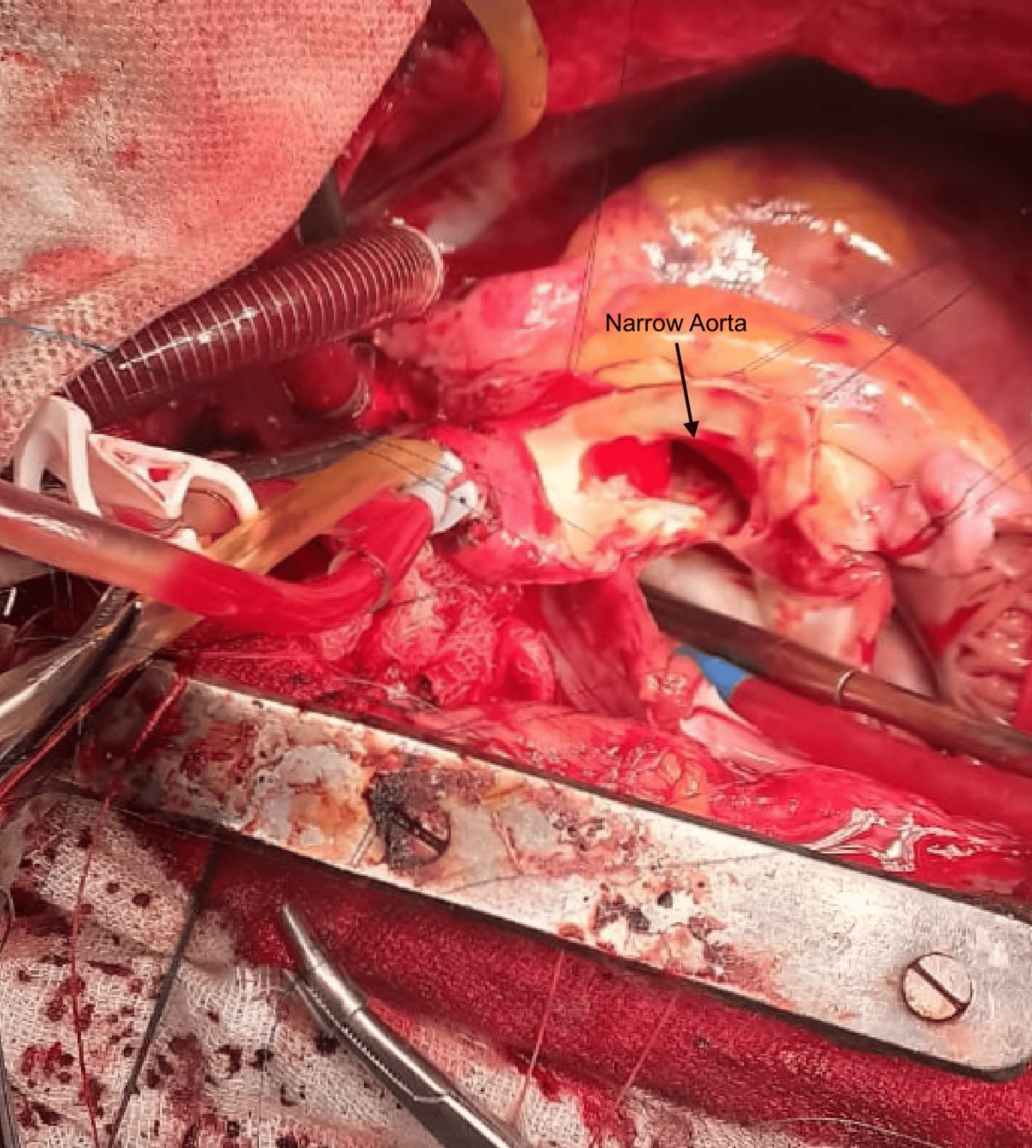
Intraoperative image showing narrow aorta and aortic root.

The surgical procedure was performed through a standard midline sternotomy approach. An autologous pericardial patch was harvested following an eccentric pericardiotomy. Intraoperatively, no right-sided SVC was identified, and the right atrial appendage was free. The left atrium was dilated. A persistent left SVC was noted, coursing posterior to the left atrium and draining into the coronary sinus, but it was missed on preoperative imaging. The right internal jugular vein central line was palpated in the innominate vein, which drained into the left SVC. An anomalous connection between the left atrium and right pulmonary artery was also observed, which was not noted in the echocardiography report. Cannulation of the aorta, left SVC, and inferior vena cava was completed, and cardiopulmonary bypass (CPB) was initiated under moderate hypothermia. Cold del Nido cardioplegia was administered partly through the aortic root and partly through direct ostial infusion following a transverse aortotomy. The non-coronary cusp leaflet was thickened. The subaortic membrane was excised, and the aortic leaflets were removed. Due to the small aortic annulus, root enlargement was deemed necessary (Figure 1).

**Figure 2:**
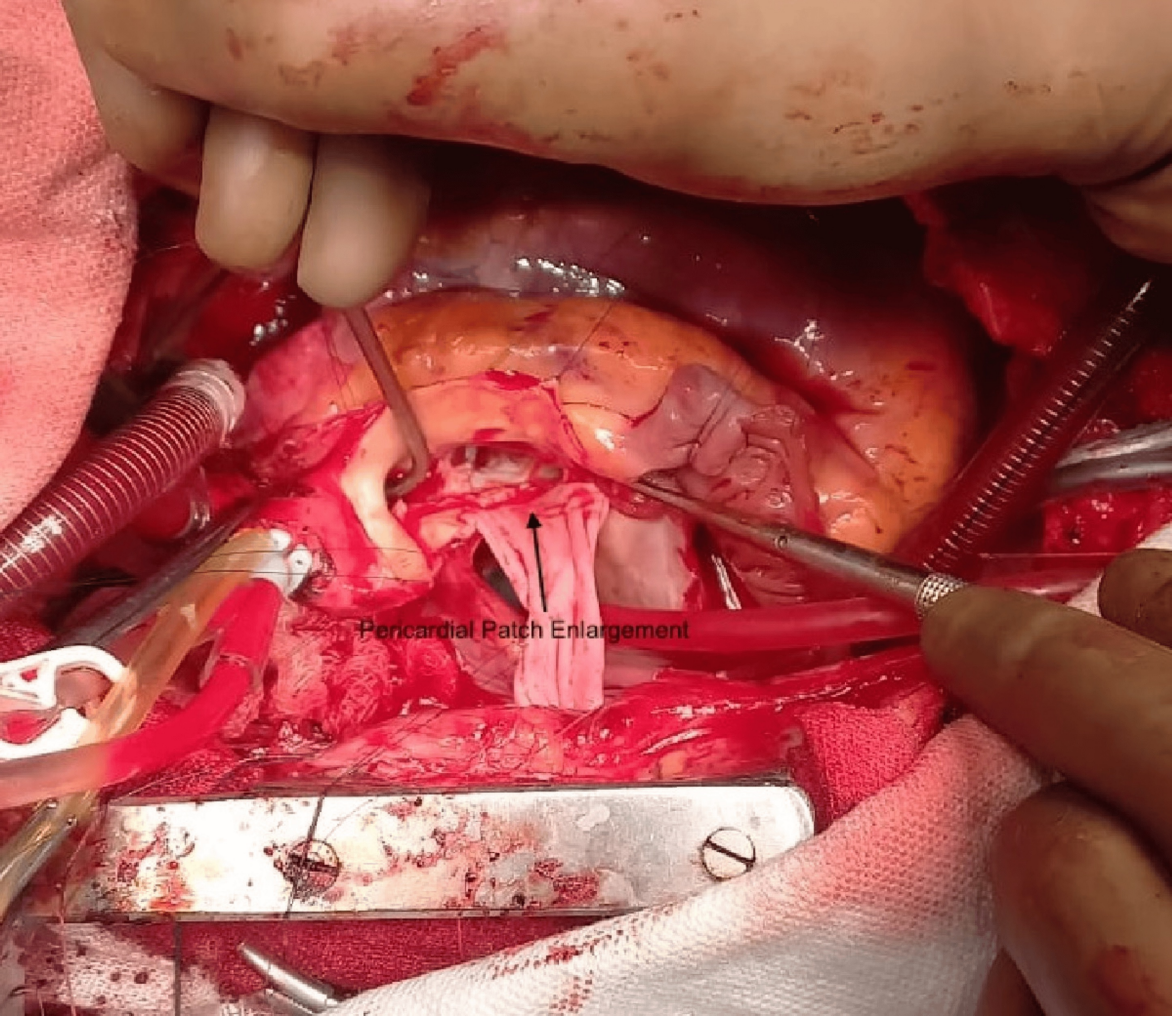
Intraoperative image showing Manouguian technique of aortic root enlargement using autologous untreated pericardial patch.

Manouguian aortic root enlargement was performed by extending the aortotomy through the annulus at the non-coronary-left coronary cusp junction and into the anterior mitral leaflet. An untreated autologous pericardial patch was used to augment the annulus (Figure 2). A Cardiamed mechanical bileaflet prosthesis, size 19, was implanted for aortic valve replacement. The mitral valve was accessed via a transseptal approach, with the incision extended into the roof of the left atrium. The supramitral ring was excised, and a saline load test showed no mitral regurgitation. The mitral valve annulus was of adequate size, and the leaflets appeared normal; therefore, no further intervention was performed. The left atrial roof was closed using a pericardial patch. The tricuspid annulus was found to be dilated, prompting a posterior leaflet plication. A subsequent saline test confirmed satisfactory valve competence. The right atrium was closed with a pericardial patch, and the direct anomalous communication between the left atrium and right pulmonary artery was ligated. The patient was weaned off CPB, with restoration of normal sinus rhythm. The remainder of the procedure was uneventful, and minimal inotropic support was required. Intraoperative transesophageal echocardiography demonstrated normal function of the bileaflet aortic prosthesis and no mitral regurgitation, with a gradient of 7 mmHg. The patient was extubated 6 hours after surgery and had an uneventful postoperative recovery despite the preoperative LV systolic dysfunction and pulmonary hypertension due to proper surgery, prosthetic valvular function, and medical management. He was discharged on postoperative day six.

## Discussion

Dr. John D. Shone initially described Shone complex as a constellation of sequential obstructions affecting both the inflow and outflow tracts of the left ventricle (LV). These include a supravalvular ring of the left atrium (also referred to as a supravalvular mitral valve ring or membrane), left ventricular outflow tract obstruction, and coarctation of the aorta [1]. The disease process begins with impaired LV inflow, which contributes to underdevelopment of the left ventricular cavity [5]. As the condition progresses, various lesions may develop, including subvalvular, valvular, or supravalvular aortic stenosis, hypoplasia of the aortic arch, and coarctation of the aorta. These features collectively define the complete form of the Shone complex [1]. The incomplete form is more common and is characterized by the presence of an LV inflow lesion, such as congenital mitral stenosis, a parachute mitral valve, or a supravalvular mitral ring, in combination with at least one LV outflow tract lesion, such as subvalvular aortic stenosis, a bicuspid aortic valve, hypoplasia of the ascending aorta, or coarctation [6]. In Shone’s original cohort, only two of eight patients exhibited the complete form, indicating the rarity of the full syndrome. Thus, awareness of incomplete variants, particularly in adult patients, remains essential and is primarily informed by case reports [5,7,8]. The supravalvular mitral membrane results from incomplete separation of endocardial cushion tissues. It typically presents as a circumferential ridge of connective tissue located on the atrial side of the mitral valve. This membrane forms a fibrous shelf superior to the mitral valve, but does not adhere to it. Its position is inferior to the left atrial appendage, distinguishing it from the membrane observed in cor triatriatum, which lies superior to the appendage. The remainder of the mitral valve apparatus is generally normal in these cases [9]. Obstruction of the LV outflow tract most commonly arises from a subaortic membrane, representing a fixed anatomic lesion. Morphologic variants of this membrane include thin, discrete endocardial folds composed of fibrous tissue; a thick fibromuscular ridge with a muscular base originating from the interventricular septum; a diffuse tunnel-like narrowing of the LV outflow tract; or, in some instances, anomalous or accessory mitral valve tissue contributing to obstruction [9].

PLSVC is the most frequently encountered congenital thoracic venous anomaly, with an estimated prevalence of 0.3-0.5% in the general population [10]. During embryological development, the thoracic venous system comprises the following two major veins: the superior cardinal veins, which drain the cranial portion of the embryo, and the inferior cardinal veins, which drain the caudal portion. These structures converge to form the right and left common cardinal veins, which subsequently enter the heart. The innominate vein arises from an anastomosis between the right and left common cardinal veins. Normally, the caudal segment of the right superior cardinal vein develops into the right SVC, whereas the left caudal segment regresses to form the ligament of Marshall. Failure of this regression results in the persistence of a left SVC, which typically drains into the coronary sinus. The most common anatomical variant of PLSVC includes the presence of both left and right SVCs, with the innominate vein either present or absent [11]. In rare cases, regression of the right superior cardinal vein leads to the absence of the right SVC, leaving the PLSVC as the sole drainage route from the cranial venous system to the right atrium via the coronary sinus. This anomaly can function without causing any hemodynamic compromise [12,13]. In patients with Shone complex, the prevalence of PLSVC is significantly higher, estimated at approximately 21% [14]. Therefore, preoperative screening to detect the presence of a left SVC is essential for optimal surgical planning and to prevent unexpected intraoperative findings. Echocardiographic diagnostic criteria for PLSVC include (i) a dilated coronary sinus observed on two-dimensional imaging in the absence of elevated right-sided filling pressures, (ii) opacification of the coronary sinus prior to the right atrium following contrast injection into a left arm vein, and (iii) normal contrast transit with right atrial opacification preceding coronary sinus enhancement when injected from the right arm [11]. The presence of a PLSVC represents a relative contraindication for retrograde cardioplegia. In such cases, clamping the PLSVC may prevent backward flow of cardioplegia into the venous system, which would otherwise lead to inadequate myocardial protection. However, cardioplegic “steal” through accessory veins remains a potential concern [11,13].

Surgical correction of the defects associated with Shone complex should be undertaken before the onset of pulmonary hypertension. The severity of mitral stenosis is a key determinant of long-term prognosis. In its most severe form, mitral stenosis leads to significantly increased pulmonary artery pressures, which are associated with the poorest clinical outcomes [15,16]. Early diagnosis of this complex congenital anomaly is important to prevent irreversible changes that can cause permanent damage to the heart.

## Conclusions

Shone complex is a rare congenital cardiac condition characterized by diverse anatomical and clinical manifestations. The coexistence of a PLSVC with an absent right SVC introduces additional complexity in both diagnosis and surgical planning. Comprehensive preoperative evaluation, including echocardiography and CT angiography, is essential for accurate anatomical assessment. When guided by detailed imaging findings, a patient-specific surgical strategy can result in favorable outcomes, even in cases with such rare and complex anomalies.
